# Illuminating Clues of Cancer Buried in Prostate MR Image: Deep Learning and Expert Approaches

**DOI:** 10.3390/biom9110673

**Published:** 2019-10-30

**Authors:** Jun Akatsuka, Yoichiro Yamamoto, Tetsuro Sekine, Yasushi Numata, Hiromu Morikawa, Kotaro Tsutsumi, Masato Yanagi, Yuki Endo, Hayato Takeda, Tatsuro Hayashi, Masao Ueki, Gen Tamiya, Ichiro Maeda, Manabu Fukumoto, Akira Shimizu, Toyonori Tsuzuki, Go Kimura, Yukihiro Kondo

**Affiliations:** 1 Pathology Informatics Team, RIKEN Center for Advanced Intelligence Project, Tokyo 103-0027, Japan; jun.akatsuka@riken.jp (J.A.); yasushi.numata@riken.jp (Y.N.); hiromu.morikawa@riken.jp (H.M.); ktsutsum@hs.uci.edu (K.T.); ichiro@insti.kitasato-u.ac.jp (I.M.); manabu.fukumoto@riken.jp (M.F.); 2 Department of Urology, Nippon Medical School Hospital, Tokyo 113-8603, Japan; area-i@nms.ac.jp (M.Y.); y-endo1@nms.ac.jp (Y.E.); s8053@nms.ac.jp (H.T.); s9078@nms.ac.jp (T.H.); gokimura@nms.ac.jp (G.K.); kondoy@nms.ac.jp (Y.K.); 3 Department of Radiology, Nippon Medical School Hospital, Tokyo 113-8603, Japan; netti@nms.ac.jp; 4 Statistical Genetics Team, RIKEN Center for Advanced Intelligence Project, Tokyo 103-0027, Japan; masao.ueki@riken.jp (M.U.); gen.tamiya@riken.jp (G.T.); 5 Tohoku Medical Megabank Organization, Tohoku University, Miyagi 980-8575, Japan; 6 Department of Pathology, Kitasato University Kitasato Institute Hospital, Tokyo 108-8642, Japan; 7 Department of Analytic Human Pathology, Nippon Medical School, Tokyo 113-8602, Japan; ashimizu@nms.ac.jp; 8 Department of Surgical Pathology, Aichi Medical University Hospital, Aichi 480-1195, Japan; tsuzuki@aichi-med-u.ac.jp

**Keywords:** deep learning, black box, prostate cancer, MRI, pathology

## Abstract

Deep learning algorithms have achieved great success in cancer image classification. However, it is imperative to understand the differences between the deep learning and human approaches. Using an explainable model, we aimed to compare the deep learning-focused regions of magnetic resonance (MR) images with cancerous locations identified by radiologists and pathologists. First, 307 prostate MR images were classified using a well-established deep neural network without locational information of cancers. Subsequently, we assessed whether the deep learning-focused regions overlapped the radiologist-identified targets. Furthermore, pathologists provided histopathological diagnoses on 896 pathological images, and we compared the deep learning-focused regions with the genuine cancer locations through 3D reconstruction of pathological images. The area under the curve (AUC) for MR images classification was sufficiently high (AUC = 0.90, 95% confidence interval 0.87–0.94). Deep learning-focused regions overlapped radiologist-identified targets by 70.5% and pathologist-identified cancer locations by 72.1%. Lymphocyte aggregation and dilated prostatic ducts were observed in non-cancerous regions focused by deep learning. Deep learning algorithms can achieve highly accurate image classification without necessarily identifying radiological targets or cancer locations. Deep learning may find clues that can help a clinical diagnosis even if the cancer is not visible.

## 1. Introduction

Recent breakthroughs in deep learning have led to great success in the field of image classification in medicine. Esteva et al. successfully classified skin diseases at the dermatological level based on a dataset of 129,450 clinical images [1]. Walsh et al. classified interstitial pneumonia on lung computed tomography (CT) images at the level of radiologists [2]. An artificial intelligence system developed by Deep Mind was shown to diagnose more than 50 common retinal diseases, with the area under the receiver operating characteristic (ROC) curve being greater than 99% [3]. This accuracy is similar to that of ophthalmologists. Bejnodi et al. reported that the accuracy of deep learning algorithms could be equivalent to that of pathologists in detecting lymph node metastases in breast cancer [4].

Deep learning’s black box problem of medical image classifications has drawn remarkable attention worldwide [5]. The Group of Twenty (G20) in Osaka 2019 had referred to the importance of explainability in its declaration on artificial intelligence principles [6]. Deep learning explainability refers to explaining why and how a machine learning approach makes its decision. Explainability is imperative for proper clinical use of deep learning techniques that can make life-altering decisions. The explainable decisions after deep learning classifications, which can be understood by humans, would allow physicians to correct the decisions made by artificial intelligence systems. Several explainable deep learning models have been proposed recently [7,8,9]. Mnih et al. reported attention-based systems which are able to provide regions of the image that the model looks at while making a classification decision in real-time [7]. The gradient-weighted class activation mapping (Grad-CAM) technique produced visual explanations of decisions made by convolutional neural networks [8]. However, the accuracies of these models while explaining the decisions of deep-learning algorithms have not been evaluated quantitatively when applied in cancer image classification. Furthermore, whether a highly accurate image classification with deep neural networks was equivalent to them truly identifying the cancer regions has not been established.

To solve these problems, we applied an explainable deep learning model to prostate magnetic resonance (MR) images. Prostate cancer is the most commonly diagnosed malignancy among males in Western countries [10,11]. MR imaging systems are widely used as non-invasive tools for the assessment of cancer location, because their high resolution provides images with excellent anatomical features and soft tissue contrast. The prostate is a unique organ for which MR images and pathological images can be referred. Thus, in this study, we compared regions of MR images, focused by deep learning through the classification process, with cancerous locations identified by radiologists and pathologists.

## 2. Materials and Methods

### 2.1. Outline of Study Design

**Figure 1:**
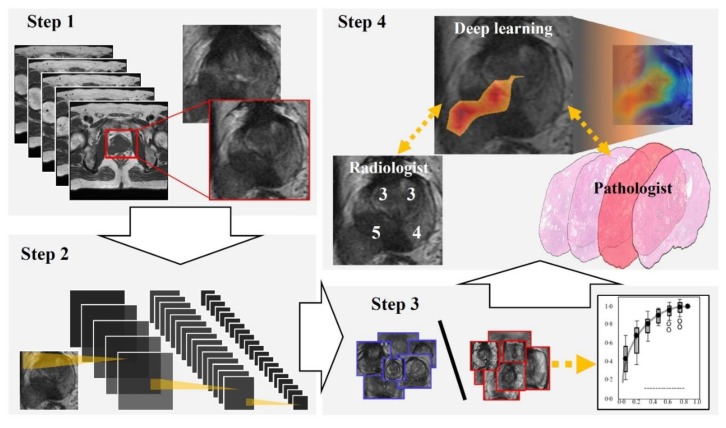
Flowchart of our study. Step 1: We extracted a rectangular region of the prostate from within the magnetic resonance (MR) images and adjusted the image size to 256 × 256 pixels. Step 2: For preparing explainable model, we applied a well-established deep neural network to MR images for cancer classification. Step 3: For evaluating the classification by the deep neural network, we constructed a receiver operating characteristic (ROC) curve with the corresponding area under the curve (AUC). Step 4: Deep learning-focused regions were compared with both radiologist-identified targets on MR images and pathologist-identified locations through 3D reconstruction of pathological images.

**Figure 2:**
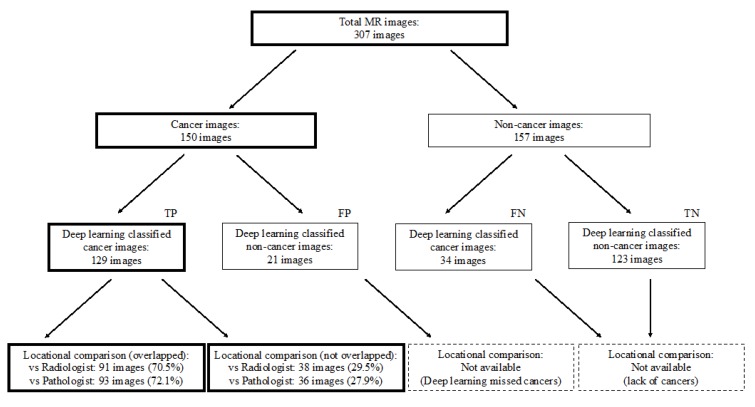
Study profile. TP = true positive, FP = false positive, FN = false negative, TN = true negative.

Figure 1 and Figure 2 show the flowchart and study profile used in this study. We used axial T2-weighted MR images for deep learning classification, which are the most popular images for deep learning analysis [12,13]. For preparing the explainable model, we conducted deep learning classification using 307 MR images after extracting a rectangular region of the prostate (Step 1 and 2 in Figure 1). We constructed an ROC curve with the corresponding area under the curve (AUC) for evaluating the classification by the deep convolutional neural network (Step 3 in Figure 1). In addition, we compared the clinicopathological features of cancer patients between the correctly classified and misclassified cases. Next, we applied an explainable deep learning model (Grad-CAM) to 129 images classified as cancer images by the aforementioned deep neural network (corresponding to the third row in Figure 2) and assessed whether the deep learning-focused regions overlapped the radiologist-identified targets based on Prostate Imaging and Reporting and Data System (PI-RADS) (Step 4 in Figure 1). Similarly, we compared the deep learning-focused regions with the pathologist-identified cancer locations through 3D reconstruction of pathological images (Step 4 in Figure 1) (Supplementary Figure S1). Finally, pathologists provided histopathological diagnoses and evaluated the deep leaning-focused regions in the non-overlapping areas.

### 2.2. Study Population

Our study included a total of 105 patients who underwent prostate MR imaging at the Nippon Medical School Hospital (NMSH) between January 2012 and May 2018. Fifty-four cases were diagnosed as PI-RADS category ≥ 3 on the MR images and diagnosed as prostate cancer after a prostate biopsy, consequently undergoing radical prostatectomy. All cancer cases included significant cancers (Gleason score ≥ 7, and/or volume ≥ 0.5 cc, and/or extraprostatic extension). Fifty-one cases were diagnosed as PI-RADS category ≤ 2 and diagnosed as benign after prostate biopsy. In this study, we excluded cases with history of prior radiation, surgery, or androgen-deprivation therapies. This study was approved by the Institutional Review Boards of NMSH (28-11-663) and RIKEN (Wako3 29-14). Informed consent was obtained from each patient.

### 2.3. MR Image Preparation

All T2-weighted MR images were saved in the format of Joint Photographic Experts Group (JPEG) files. MR images that included cancer regions were used as positive training data and MR images without cancers were used as negative training data. We extracted a rectangular region of the prostate from the image. This rectangular region included proximate tissues such as prostatic capsular vessels, pelvic fascia, and rectum (Step 1 in Figure 1). We then adjusted these images to a size of 256 × 256 pixels for deep learning analysis. For PI-RADS analysis, we used multiparametric MR images in the format prescribed by the Digital Imaging and Communications in Medicine (DICOM).

### 2.4. MR Imaging Settings

All patients underwent multiparametric MR imaging including T2-weighted, diffusion-weighted, and dynamic contrast-enhanced T1-weighted imaging before prostate biopsy. Each scan was performed using a mixed MR imaging scanner with different gradient strengths (1.5 or 3.0 tesla) with a phased array coil. A previous study had revealed that the signal-to-noise ratio and contrast noise ratio of T2 weighted imaging were similar at 1.5 and 3.0 tesla [14]. In the current study, diffusion-weighted imaging was used only for PI-RADS scoring. Note that the mixed scanners setting was clinically more realistic than the settings of other previous studies that recruited MR images from a single and specific MR imaging scanner [12,13].

### 2.5. Classification Using a Deep Neural Network (Preparation for an Explainable Deep Learning Model) 

First, we tested three deep convolutional neural network models, Xception [15], inceptionV3 [16], and VGG16 [17], that were pre-trained on ImageNet with classification layers adapted to our labels. We selected the Xception in this study because we found that the Xception showed the most precise performance for MR image classification. We used 10-fold cross-validation to test the prediction models, randomly dividing the whole cases in a 1:9 ratio, using one part for testing and the other nine parts for training [18,19]. In each split set, the test data did not include any MR images of the training data cases. Cross-validation is a basic method of comparing and evaluating the performance of a machine learning model [12]. For each testing/training split, we used the AUC metric to assess the performance of the trained prediction models on the test data using the cvAUC package of software R [20,21]. This study employed the RIKEN AIP Deep Learning Environment (RAIDEN) supercomputer system for all computations.

### 2.6. Clinicopathological Evaluations

We compared the classified and misclassified cases based on the following clinicopathological data: age, prostate-specific antigen (PSA) level, total prostate volume (TPV), PSA density (PSAD), clinical T stage, Gleason score, pathological T stage, and blood test data. We judged cases as classified or misclassified based on the most plausible prediction value of the MR images in each case.

### 2.7. Preparation of Pathology Images

Prostates after radical prostatectomy were fixed with 10% formalin. Formalin-fixed whole prostates were dissected into several approximately 3−5 mm slices in a direction perpendicular to rectum surface from the prostate apex to the bladder neck and were embedded in paraffin. All slices were further sectioned at a thickness of 3 μm and stained with hematoxylin and eosin (H&E). All H&E-stained slides were scanned by a whole-slide imaging scanner (NanoZoomer S60 Digital Slide Scanner, Hamamatsu, Japan) with a 20× objective lens and were stored on a secure computer.

### 2.8. Scoring on MR Images 

Experienced radiologists evaluated all multiparametric MR images including the T2 weighted, diffusion weighted and dynamic contrast enhanced T1 weighted images. For the scoring, each target region was categorized based on PI-RADS, which is a scoring system for standardization in prostate multiparametric MR imaging reporting [22]. The radiologists were blind to all clinicopathological information.

### 2.9. Pathological Cancer Grading

Prostate cancer was diagnosed pathologically based on the International Society of Urological Pathology (ISUP) grading [23]. Pathologists diagnosed all the cases and marked cancer locations independently and reached a collective consensus.

### 2.10. Locational Comparison between Deep Learning-Focused Regions on MR Images and Expert-Identified Cancer Locations

Through the 3D reconstruction of pathological images corresponding to MR images, we selected an appropriate pathological image closest to the MR image. We oriented these two types of images based on 8 landmark points: the location of the anterior, posterior, and bilateral-external midlines at each peripheral zone and transition zone (Supplementary Figure S1). In order to evaluate the deep learning-focused regions, we applied the Grad-CAM technique to construct saliency maps from all prostate cancer cases [8]. Grad-CAM is a technique used for producing visual explanations of decisions made by convolutional neural networks. We defined regions with Hue ≤ 0.1, in the HSV (hue, saturation, value) images of the saliency map as the deep learning-focused regions. On the other hand, radiologists evaluated multiparametric MR images that corresponded to the T2-weighted MR images and provided scores for each of the 12 divided regions based on PI-RADS: bilateral peripheral zone and transition zones located at the apex, mid, and base of the gland. The regions of the MR images with PI-RADS scores higher than or equal to 4 were considered as radiologist-identified targets. We counted the number of images with overlapping areas between the deep learning-focused regions and the radiologist-identified targets using an automated algorithm. Finally, we also counted the number of images with overlapping areas between the deep learning-focused regions and the pathologist-identified cancer locations using an automated algorithm.

### 2.11. Statistical Analysis

We compared the patient characteristics of the cancer and non-cancer cases and clinicopathological characteristics of the classified and misclassified cancer cases using the Wilcoxon test for continuous data and the Fisher’s exact test for categorical data. We conducted a hypothesis test for a population proportion (null proportion is 0.5) in order to examine the matching rate between deep learning-focused regions and expert-identified cancer locations. All reported P values were two-sided with the level of statistical significance set at *p* < 0.05. We used the JMP software version 13.0 for the statistical analyses

## 3. Results

### 3.1. Image and Patient Characteristics

Table 1 summarizes the patient characteristics of this study. The mean age was 67.4 and 65.2 for the cancer and the non-cancer cases (*p* = 0.09), respectively, while the serum PSA level for the cancer cases was significantly higher than that for the non-cancer cases (mean: 14.7 vs. 8.1 ng/mL, *p* < 0.001). Further, the TPV was significantly lower in the cancer cases than in the non-cancer cases (mean: 27.5 vs. 42.5 mL, *p* < 0.001), while the PSAD was significantly higher in the cancer cases (mean: 0.63 vs. 0.22 ng/mL/cm^3^, *p* < 0.001).

### 3.2. Classification Using a Deep Neural Network (Preparation for an Explainable Deep Learning Model) 

**Figure 3:**
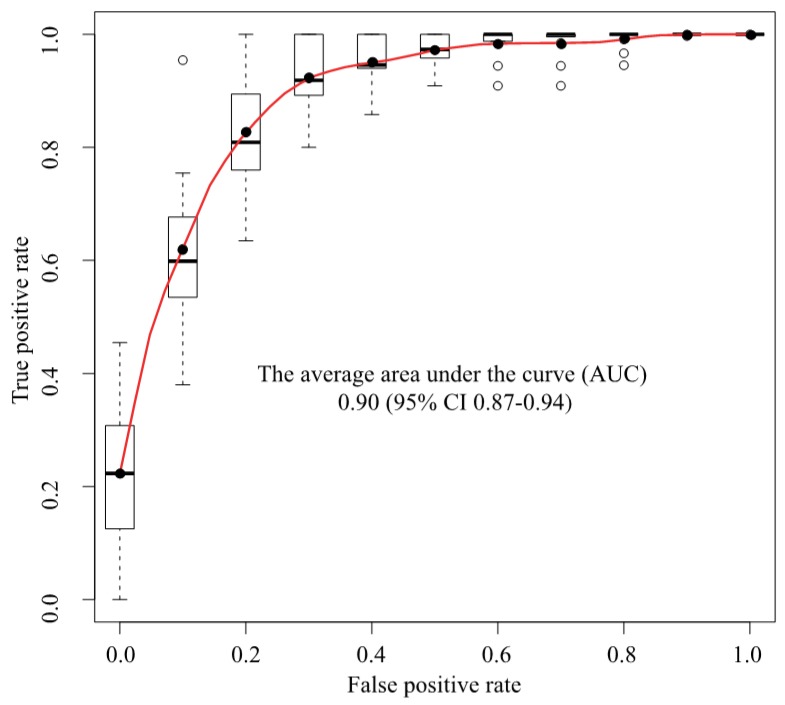
ROC analysis. The average AUC was 0.90 (95% CI 0.87–0.94). ROC = Receiver operating characteristics, AUC = Area under the curve, CI = Confidence interval, Black circle = Average, White circle = Out of range value, Black solid line = Median, Box = Interquartile range, Dashed line = Range, Upper black line = Maximum value, Bottom black line = Minimum value.

We used a deep convolutional neural network (Xception) to classify 307 MR images as either cancer or non-cancer cases. We constructed an ROC curve for classification accuracy using 10-fold cross validation (Figure 3), which yielded an average AUC of 0.90 (95% confidence interval (CI) 0.87–0.94). Supplementary Figure S2 shows the case-level analysis for an average AUC of 0.93 (95% CI 0.87–0.99). In the case of the cancer images, 86.0% of the images were classified correctly, whereas 14.0% were misclassified. In the case of the non-cancer images, 78.3% were classified correctly, whereas 21.7% were misclassified.

### 3.3. Clinical Comparison of Cases Classified Using a Deep Neural Network

We compared the clinicopathological features of the cancer and non-cancer cases classified by the deep convolutional neural network (Table 2). The Gleason score was higher in the misclassified cases than in the classified cases (*p* = 0.03). There were no significant differences between the classified and misclassified cases with respect to age, PSA, TPV, PSAD, clinical T stage, pathological T stage, and other blood test data.

### 3.4. Locational Comparison between Deep Learning-Focused Regions on MR Images and Expert-Identified Cancer Locations

**Figure 4:**
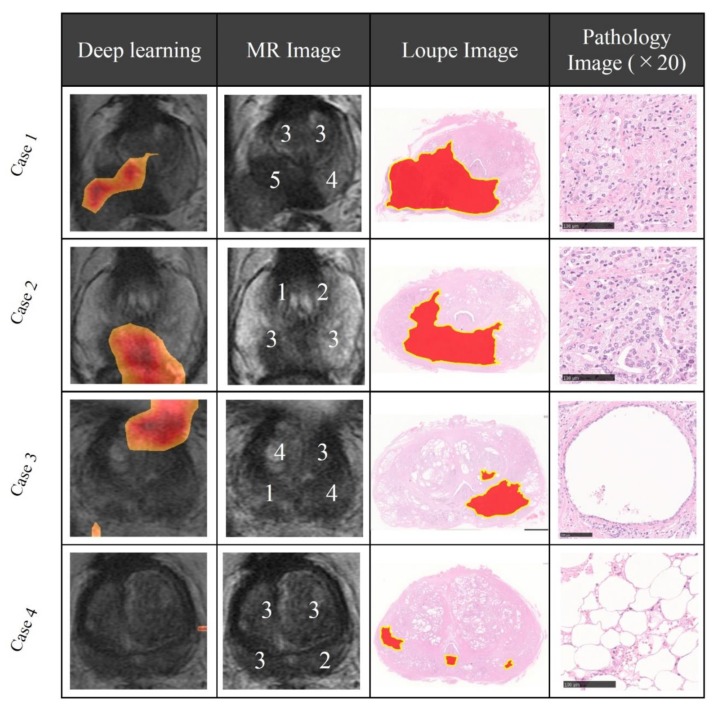
Representative cases of deep learning-focused regions and expert-identified cancers. Left: Deep learning focused regions. Second left: MR image with PI-RADS score. Second right: Loupe image of pathology slides (red area indicates cancer locations). Right: Representative pathological image in the deep learning-focused region (×20). Details of each cases are shown in Supplementary Table S1. MR image = Magnetic resonance image, PI-RADS = Prostate imaging reporting and data system.

**Figure 5:**
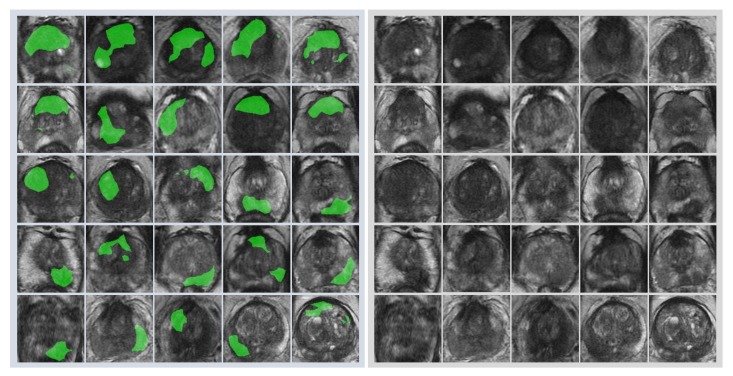
Representative images with overlapping areas between deep learning-focused regions and genuine cancer locations. Left image group: 25 images with overlapping areas between deep learning-focused regions and genuine cancer locations. The overlapped areas are colored in green. Right image group: corresponding 25 raw MR images. MR images = Magnetic resonance images.

First, radiologists evaluated multiparametric MR images that corresponded to the T2-weighted MR images analyzed by deep learning algorithms based on PI-RADS. We locationally compared the deep learning-focused regions of the MR images with the radiologist-identified targets (PI-RADS category ≥ 4). We found that the deep learning-focused regions overlapped the radiologist-identified targets in 70.5% of the MR images (*p* < 0.001). Next, the locational correlation between the deep learning-focused regions in the MR images and genuine cancer locations based on 3D reconstruction of pathological images was evaluated. We found that the deep learning-focused regions overlapped genuine cancer locations in 72.1% of the MR images (*p* < 0.001). In the remaining MR images, deep learning focused the following regions: transition zone (10.1%), peripheral zone (7.8%), and the others (region outside of prostate gland). Figure 4 and Supplementary Table S1 show four representative cases of the comparative analyses. In cases one and two, deep learning focused on correct cancer regions. On the other hand, in case three, deep learning focused on a non-cancerous region. In case four, deep learning focused on normal adipose tissue. Figure 5 shows 25 representative images with overlapping areas between deep learning-focused regions and genuine cancer locations. The overlapped areas are colored in green. Finally, pathologists evaluated the deep leaning-focused regions in the non-overlapping images and found that deep learning focused on following regions: dilated prostatic ducts, lymphocyte aggregation, and others (normal stroma and adipose tissue) (Supplementary Figure S3).

## 4. Discussion

We elucidated the differences between deep learning and human approaches in cancer image classifications using an explainable deep learning model. First, prostate MR images were classified using a deep neural network without locational information of cancers. Wang et al. reported an AUC of 0.84 for their deep learning classification using prostate cancer MR images [12]. In this study, we reported an AUC of 0.90 using the latest deep learning techniques. Subsequently, we assessed the clinicopathological features of cancer cases classified and unclassified by deep learning analysis. Interestingly, we found significantly higher Gleason score in the misclassified cases, when compared to the correctly classified cases. The Gleason score is one of the most important predictors of prostate cancer prognosis [24]. A higher Gleason score (≥ 8) implies greater tumor aggressiveness. It may reflect these pathological features on MR images [25,26].

Next, we quantitatively analyzed the correlation between the deep learning-focused regions in the MR images and the expert-identified cancers. In this study, radiologists identified targets on MR images based on PI-RADS, which is an accurate tool for the detection of clinically significant prostate cancers and which has been developed for standardizing the interpretations of prostate MR imaging [22]. Deep learning-focused regions overlapped the radiologist identified cancer targets (PI-RADS category ≥ 4) by 70.5%. Furthermore, we compared the deep learning-focused regions with pathologist identified cancer locations. For comparing the MR images and pathological images, we created 3D reconstructions of pathological images of the prostate. The deep learning-focused regions overlapped the pathologist identified cancer locations by 72.1%.

There are several possible reasons for the discrepancy between deep learning-focused regions and expert-identified locations. Deep learning may be able to identify cancer-related features in non-cancerous regions. Our results propose dilated prostatic duct and lymphocyte aggregation as possible cancer-related features of prostate cancers. Although inflammation in carcinogenesis of prostate is still controversial, the relationship between the inflammatory microenvironment and prostate cancer progression have been reported [27]. Furthermore, Miyai et al. reported that luminal spaces could be considered one of the predictors in the in vivo MR imaging-detectability of prostate cancer [28]. These findings are consistent with the result from our deep learning analysis. The second possibility is that these cases could have been the result of overfitting of deep neural networks. Overfitting is a problem that occurs when the algorithms fit the training data too closely instead of generalization, which is the most important cautionary factor related to machine learning. There are several methods for reducing overfitting in deep learning classification [29,30]. A dropout method can effectively reduce overfitting in deep neural networks [29]. Such an algorithm drops some connections between neural networks randomly. Data augmentation is another way to address overfitting [30]. Using this method, we can increase the training data by applying a transformation. In this study, we employed data augmentation for cancer image classification on MR images. Deep learning may find not only un-generalized features outputted through its overfitting to the limited dataset but also genuine medical clues that can help a clinical diagnosis by a physician even if the cancer is not visible in the region, indicating the high potential utility of deep learning in the field of image diagnosis.

The main limitation of this study is that it was conducted using the cross-validation method in a single facility. However, the purpose of this study was to compare between deep learning-focused regions and expert-identified cancers. Furthermore, our study provides robust results because our dataset is one of the largest pathological 3D reconstruction datasets of a prostate corresponding to MR images as far as we know.

In summary, we elucidated the differences between deep learning and expert approaches for identifying cancer on MR images. We found that deep learning algorithms could achieve highly accurate image classification without necessarily identifying radiological targets or cancer locations. Deep learning may find a clue that can facilitate an imaging diagnosis even if the cancer is not visible in the region, beyond the overfitting. Deep learning technology is an attractive and useful tool that can assist physicians and improve patient health. For effective utilization of this technology, we must pursue the medical meanings behind deep learning classifications.

## 5. Conclusions 

Our study provides a new paradigm for searching cancer-related features on MR images using an explainable deep learning model. We succeeded in elucidating the difference of cancer-detection approaches between those taken by human and deep learning through applying the explainable model to pathological 3D reconstruction corresponding to MR images. MR imaging systems are non-invasive tools for providing high resolution images and information of cancer locations. On the other hand, pathological examination is an invasive method but provides definitive diagnoses. Our study opens the door for facilitating MR image analysis using an explainable model based on pathological evidence and contributes to clinicians′ understanding and management of deep learning for its medical use in the near future. Deep learning is a tool with high potential not only for cancer image classifications but also for discovering clues of cancer that have not been known to humans.

## Figures and Tables

**Table 1:** Patient characteristics PSA = Prostate-specific antigen, TPV = Total prostate volume, PSAD = PSA density, SD = Standard deviation.

Total Cases: N = 105	Cancer Cases	Non-Cancer Cases	*p* Value
Number of cases, n	54	51	-
Age, year, mean ± SD	67.4 ± 6.9	65.2 ± 8.6	0.09
PSA, ng/mL, mean ± SD	14.7 ± 12.1	8.1 ± 5.4	<0.001
TPV, mL, mean ± SD	27.5 ± 10.6	42.5 ± 19.3	<0.001
PSAD, ng/mL/cm^3^, mean ± SD	0.63 ± 0.66	0.22 ± 0.16	<0.001

**Table 2:** Univariate analysis of clinicopathological features: deep learning classified cancer cases versus misclassified cancer cases. PSA = Prostate-specific antigen, TPV = Total prostate volume, PSAD = PSA density, WBC = White blood cell, Hb = Hemoglobin, Plt = Platelet, LDH = Lactate dehydrogenase, ALP = Alkaline phosphatase, Ca = Calcium, SD = Standard deviation.

Cancer Cases: N = 54	Classified Cases	Misclassified Cases	Univariate (*p* Value)
Number of cases, (%)	92.6	7.4	
Age, years, mean ± SD	67.4 ± 6.9	67.5 ± 7.3	0.96
PSA, ng/mL, mean ± SD	14.2 ± 11.9	21.6 ± 13.5	0.07
TPV, mL, mean ± SD	27.9 ± 10.7	23.0 ± 10.2	0.66
PSAD, ng/mL/cm^3^, mean ± SD	0.59 ± 0.64	1.17 ± 0.85	0.07
Gleason score, (%)			0.03
<8	60.0	0.0	
≥8	40.0	100.0	
Clinical stage, (%)			0.21
≤T2	80.0	50.0	
≥T3	20.0	50.0	
Pathological stage, (%)			0.63
≤T2	44.0	25.0	
≥T3	56.0	75.0	
WBC, 10^3^/μL, mean ± SD	6074 ± 1248	5150 ± 656	0.12
Hb, g/dl, mean ± SD	14.5 ± 1.2	13.8 ± 0.7	0.08
Plt, 10^3^/μL, mean ± SD	21.8 ± 5.0	18.3 ± 2.5	0.14
LDH, U/L, mean ± SD	180 ± 34.9	179 ± 45.4	0.93
ALP, U/L, mean ± SD	208 ± 56	249 ± 161	0.75
Ca, mg/dL, mean ± SD	9.3 ± 0.43	9.1 ± 0.26	0.29

## Supplementary Materials

The following are available online at https://www.mdpi.com/2218-273X/9/11/673/s1, Figure S1: 3D reconstruction of pathological images corresponding to MR images Through 3D reconstructions of pathological images, we selected appropriate pathological images closest to the MR images and then oriented these two types of images based on 8 landmark points. MR images = Magnetic resonance images, Figure S2: ROC analysis of case-level classification. The average AUC was 0.93 (95% CI 0.87–0.99). ROC = Receiver operating characteristic, AUC = Area under the curve, CI = Confidence interval, Black circle = Average, White circle = Out of range value, Black solid line = Median, Box = Interquartile range, Dashed line = Range, Upper black line = Maximum value, Bottom black line = Minimum value, Figure S3: Pathological findings. Dilated prostatic duct and lymphocyte aggregation were observed in the pathological images at the locations focused by deep learning, Table S1: Detailed characteristics of representative cases. PSA = Prostate-specific antigen, MR image = Magnetic resonance image.
