# Supplementary material for: Illuminating Clues of Cancer Buried in Prostate MR Image: Deep Learning and Expert Approaches
Source: Biomolecules. 2019 Oct 30;9(11):673. doi: 10.3390/biom9110673 (PMC6920905; doi:10.3390/biom9110673)
Supplement: Supplementary file 1 [file biomolecules-09-00673-s001.pdf]

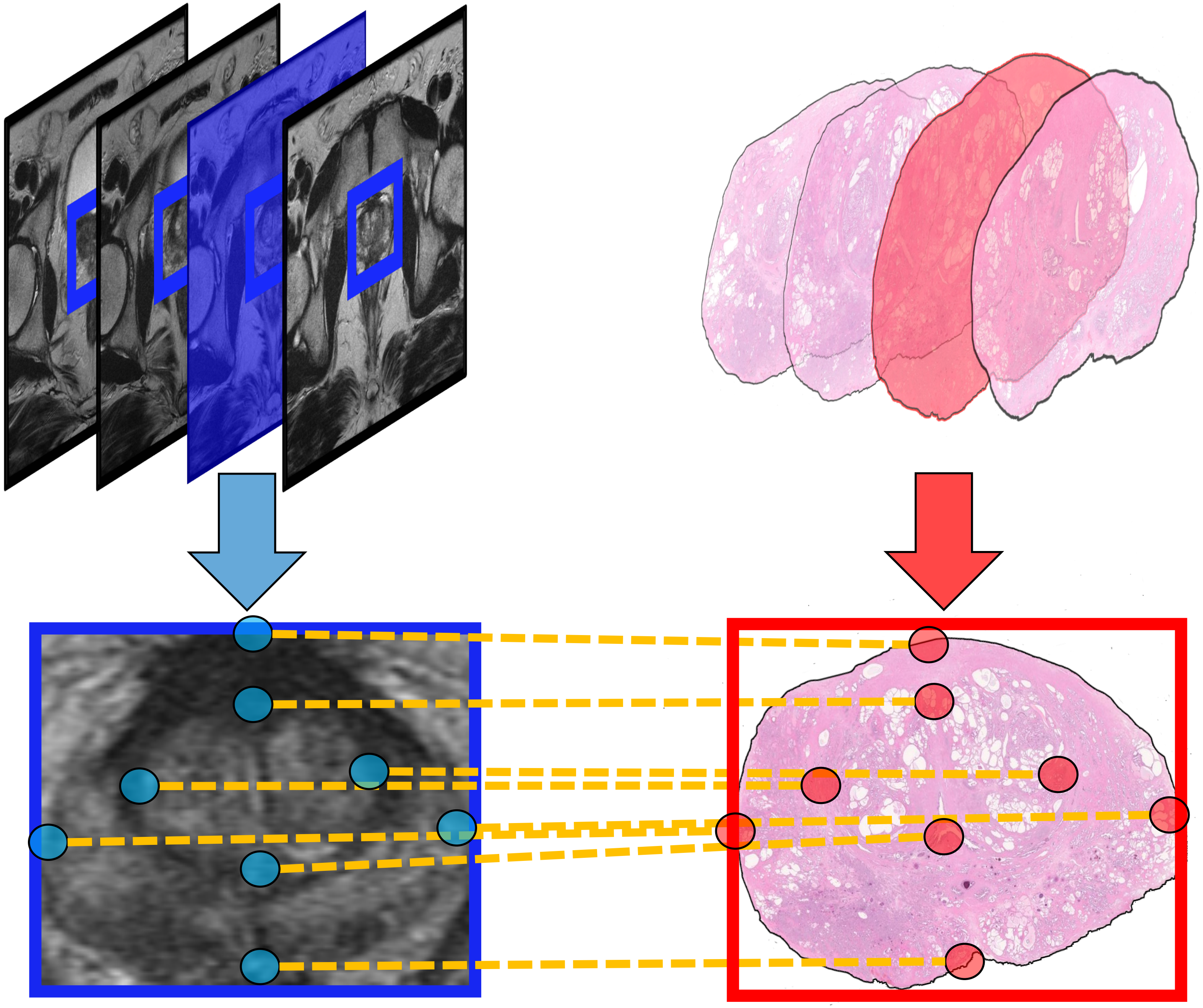

**Supplementary Figure S1. 3D reconstruction of pathological images corresponding to MR images**

Through 3D reconstructions of pathological images, we selected appropriate pathological images closest to the MR images and then oriented these two types of images based on 8 landmark points.
MR images = Magnetic resonance images

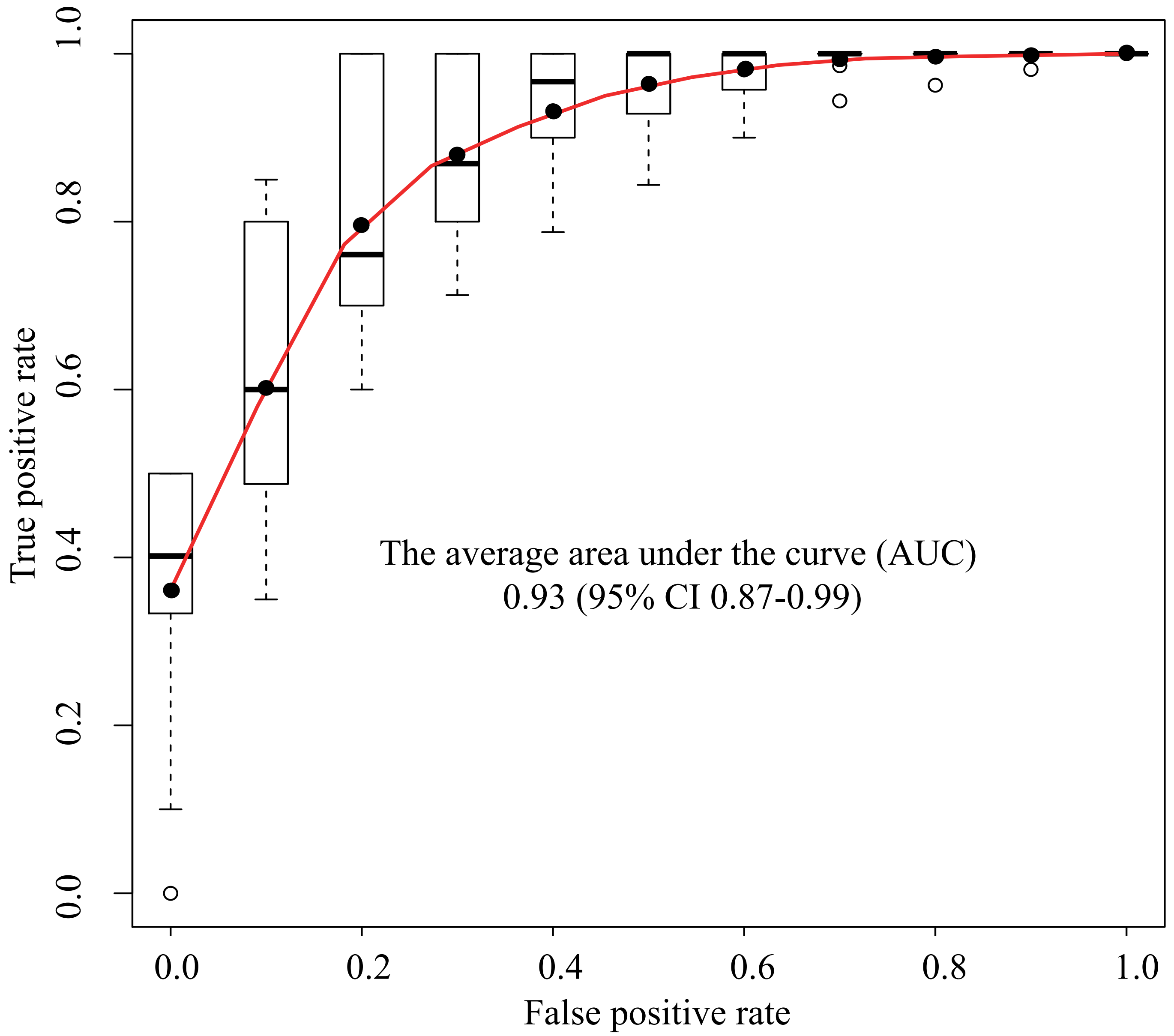


**Supplementary Figure S2. ROC analysis of case-level classification**

The average AUC was 0.93 (95% CI 0.87-0.99).
ROC = Receiver operating characteristic, AUC = Area under the curve, CI = Confidence interval, Black circle = Average, White circle = Out of range value, Black solid line = Median, Box = Interquartile range, Dashed line = Range, Upper black line = Maximum value, Bottom black line = Minimum value

Dilated prostatic duct

lymphocyte aggregation

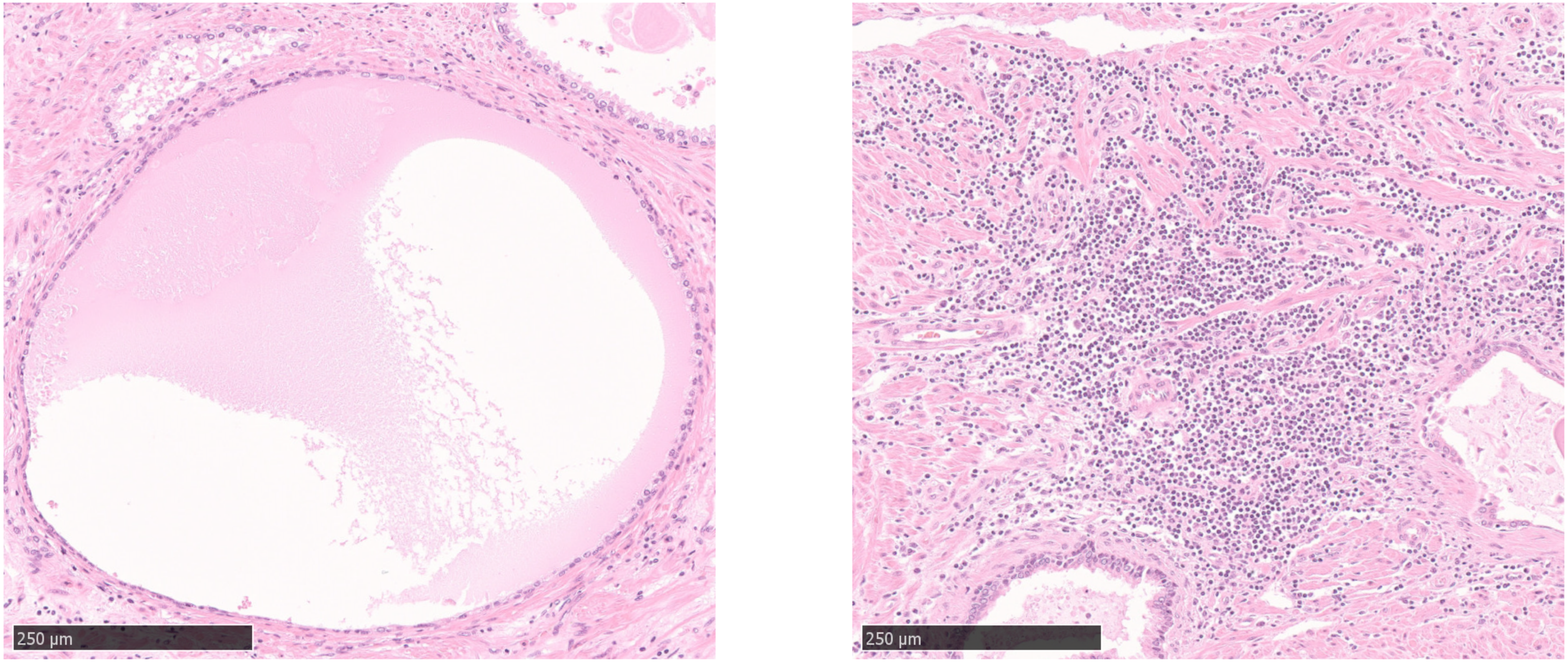


**Supplementary Figure S3. Pathological findings**

Dilated prostatic duct and lymphocyte aggregation were observed in the pathological images at the locations focused by deep learning.

The epoch was set to 20 and batch size was set to 4. Keras's ImageDataGenerator was used for image augmentation using the following parameters: featurewise_center = False, samplewise_center = False, featurewise_std_normalization = False, samplewise_std_normalization = False, zca_whitening = False, rotation_range = 0.0, width_shift_range = 0.01, height_shift_range = 0.01, shear_range = 0.0, zoom_range = 0.01, channel_shift_range = 0.0, fill_mode = 'nearest', cval = 0.0, horizontal_flip = True, vertical_flip = False, rescale = 1.0/ 255.0

**Supplementary Table S1. Hyper parameters of the deep learning algorithms for image classification**

**Case 1**

Sixty-one-year-old male with serum PSA10.8 ng/ml. T2 weighted MR image showed homogeneous low signal intensity in the posterior peripheral zone on the right in the posterior-portion of the prostate. This abnormal signal intensity was identified as prostate cancer with a Gleason score of 9 (5 + 4) with prostate capsular invasion on the pathological image. Deep learning focused on the cancer region.

**Case 2**

Fifty-eight-year-old male with serum PSA16.0ng/m. T2 weighted MR image showed homogeneous low signal intensity in the posterior peripheral zone in the mid-portion of the prostate. This abnormal signal intensity was diagnosed as prostate cancer with a Gleason score of 9 (4 + 5) on the pathological image. Deep learning focused on the cancer region.

**Case 3**

Fifty-year-old male with serum PSA9.2 ng/ml. Deep learning focused on a region in the anterior transitional zone where no tumor was present on the pathological image. Instead, dilated prostatic ducts were observed in the deep learning-focused region. The pathological image showed prostate cancer with Gleason score 7 (3 + 4) mainly in the posterior peripheral zone on the left.

**Case 4**

Seventy-four-year-old male with serum PSA8.1 ng/ml. T2 weighted MR image showed diffuse and heterogeneous low signal intensity in the transitional and peripheral zone of the prostate. The pathological image showed prostate cancer with Gleason score 7 (4 + 3) in the posterior peripheral zone. Deep learning focused on normal adipose tissue in the extra prostatic region, which might be a un-generalized feature outputted due to overfitting.

**Supplementary Table S2. Detailed characteristics of representative cases**

PSA = Prostate-specific antigen, MR image = Magnetic resonance image
